# Functional Characterization of Ammonium Transporter *LjAMT2;4* During *Lotus japonicus* Symbiosis with Rhizobia and Arbuscular Mycorrhizal Fungi

**DOI:** 10.3390/jof11050340

**Published:** 2025-04-27

**Authors:** Kailing Xie, Ying Ni, Lijie Bai, Yuqian Zhai, Wenqing Zhou, Beijiu Cheng, Xiaoyu Li

**Affiliations:** Key Laboratory of Crop Stress Resistance and High-Quality Biology of Anhui Province, Anhui Agricultural University, Hefei 230036, China

**Keywords:** rhizobia, arbuscular mycorrhizal fungi, ammonium transporter, symbiosis, *Lotus japonicus*

## Abstract

Arbuscular mycorrhizal fungi (AMF) and rhizobia are important symbiotic microorganisms in soil, which can symbiose with legumes to form mycorrhizal symbionts and nodules, respectively. Once a stable symbiotic relationship is established, these microorganisms have been found to enhance nitrogen absorption by legumes. Although plants can directly utilize ammonium through ammonium transporters (AMTs), there is limited research on the role of the AMT gene family in promoting ammonium transport in symbiotic relationships. *Lotus japonicus*, a common host of arbuscular mycorrhizal fungi and rhizobia, serves as a model legume plant. In this study, we examined the characteristics of the ammonium transporter *LjAMT2;4* in *L. japonicus* and found that *LjAMT2;4* is localized to the plasma membrane and is predominantly expressed in roots. The promoter region of *LjAMT2;4* contains cis-acting elements induced by arbuscular mycorrhizal fungi and rhizomes, and the expression of *LjAMT2;4* was induced by AM fungi and rhizobia. However, there was no significant difference in the mycorrhizal colonization rate of *ljamt2;4* compared to the wild type, while the absence of *LjAMT2;4* significantly increased the number of root nodules under nitrogen-starved conditions, enhancing nitrogen fixation and alleviating nitrogen stress in extremely nitrogen-starved environments, ultimately promoting plant growth. These findings suggest that manipulating the genes involved in symbiotic nitrogen fixation, such as *LjAMT2;4*, could offer new strategies for sustainable agricultural production. Given that AM and rhizobia symbiosis are critical for crop growth, our findings may inform strategies to improve agricultural management.

## 1. Introduction

Nitrogen (N) is a crucial mineral nutrient that is essential for plant growth and metabolism. Nitrogen deficiency in plants can result in a dwarf plant phenotype with yellowing leaves [1,2]. To cope with N-deficient stress, plants must evolve several defense mechanisms, such as enhancing N transport through root–soil interface expansion and forming symbiotic relationships with rhizosphere microorganisms [3].

Arbuscular mycorrhizal fungi (AMF) and nitrogen-fixing rhizobia are two well-studied beneficial microbes that form symbiotic relationships with host plants [4]. Most terrestrial plants participate in a mutually beneficial endosymbiotic relationship with arbuscular mycorrhizal (AM) fungi, enhancing their ability to obtain essential mineral nutrients from the soil, particularly phosphorus and nitrogen [5]. In exchange, host plants furnish AM fungi with the organic carbon essential for their sustenance and propagation [6]. In legumes, N can be absorbed from the soil through arbuscular structures in symbiosis with AM fungi and from the air through nodular tissue in symbiosis with rhizobia. According to the statistics, nitrogen fixation accounts for 65% of biological nitrogen fixation in agricultural systems [7].

The promotion of the plant uptake of ammonium nitrogen through symbiosis is primarily regulated by specific genes, with ammonium transporters (AMTs) on the cytoplasmic membrane playing a key role in the regulation of the relevant genes and acting as the main responsible factors for transporting ammonium nitrogen in plants [8]. Ammonium nitrogen transporter proteins in angiosperms are classified into two families (AMT1 and AMT2), with an average of five members in each family [9]. For example, *SbAMT3;1*, *OsAMT1;1*, and *TaAMT1;2* in sorghum, rice, and wheat are significantly induced in arbuscular roots [10]; *MtAMT2;2* and *AtAMT1;3* are specifically upregulated during mycorrhizal symbiosis; *GmAMT4.1* in soybean and *SbAMT3.1* in sorghum [11,12,13] have been shown to be localized on the peri-arbuscular membrane; and *SbAMT4* can amplify ammonium ion-deficient yeast mutants. *LeAMT4* and *LeAMT5* in tomato are only expressed in mycorrhizal roots [14], and the transcript abundances of *PtAMT1.2*, *PtAMT1.2b*, *PtAMT1.3*, *PtAMT1.4a*, and *PtAMT1;2* in poplar are also strongly induced by mycorrhizal formation [15]. In conclusion, one or more ammonium transporters have been found to be specifically induced or significantly upregulated in expression by AM fungi in *Medicago truncatula* Gaertn., *Centella asiatica* (L.) Urb., *Glycine max* (L.) Merr., and *Sorghum bicolor* (L.) Moench. [12,13,16,17], mainly the AMT2 subfamily induced by AM fungi. These studies suggest that, at least in these species, these transporters likely play a role in the transport of ammonium ions across arbuscular membranes.

The symbiotic relationship between legumes and rhizobia involves several key processes, including recognition, infection, nodulation, and nitrogen fixation [18,19,20]. There are two modes of nodule formation: the invasion of root hairs and the direct invasion of the root through the destruction of the epidermis [21]. Leguminous symbiosis is the first mode. Leguminous plants form symbiotic relationships with rhizobia, which can convert atmospheric nitrogen into nitrogenous compounds directly usable by plants, thus significantly reducing the nitrogen requirements of these plants. In return, legumes provide carbohydrates and mineral nutrients to rhizobia [22,23]. The establishment of symbiotic nitrogen fixation relies on mutual recognition between plants and *Rhizobium* [19]. Under nitrogen deficiency, legume roots release flavonoids into the inter-root space, which induces the secretion of nodule factors by rhizobia. Legume roots induce root hair curling, invagination line formation, and cortical cell division upon sensing signals from nodule factors, inducing root nodule morphogenesis [24]. The legume cell membrane receptor-like kinases *NFR1* (Nod Factor Receptor 1) and *NFR5* recognize the nodulation factors secreted by rhizobia to initiate symbiotic signaling. However, the molecular mechanisms by which symbiotic signals are transmitted downstream of the membrane receptors are less well studied [25]. In *L. japonicus*, a gene named *SYMRKL1* encodes a protein with a domain nearly identical to *SYMRK*, which is required for normal infestation line formation [26]. Therefore, we aimed to determine the process it undergoes, from the formation of infection threads to the generation of nodules.

The legume *L. japonicus* is a valuable model crop for studying both AMF symbiosis and rhizobia symbiosis. While significant research has been conducted on symbiotic ammonium nitrogen transporter genes in other species, such as sorghum and rice, less is known about the function of mycorrhizal-inducible nitrogen transporter genes in *L. japonicus* compared to the extensive research on symbiotic ammonium nitrogen transporter genes in sorghum and rice. In the present work, the characteristic analyses of *LjAMT2;4* indicate that *LjAMT2;4* is a typical ammonium transporter, and that it is localized in the plasma membrane and mainly expressed in roots. Additionally, there are both arbuscular mycorrhizal-induced and rhizome-induced cis-acting elements in its promoter region. Although *LjAMT2;4* was induced by AM fungi, there was no significant difference in the mycorrhizal colonization rate of *ljamt2;4* compared to the wild type. However, the absence of *LjAMT2;4* significantly increased the number of root nodules under different nitrogen-starved environments and effectively fixed nitrogen in the air, thus alleviating nitrogen stress in extremely nitrogen-starved environments and promoting plant growth. At the same time, because *LjAMT2;4* itself does not have the ability to transport ammonium nitrogen, the nodular nitrogen fixation it develops is beneficial for the plant’s nitrogen absorption. These findings offer insights into the potential of genes to regulate symbiotic nitrogen fixation and present novel strategies for sustainable agricultural development.

## 2. Materials and Methods

### 2.1. Bioinformatics Analysis of AMT Genes

To investigate the phylogenetic connections among AMT proteins in *Arabidopsis thaliana* (L.) Heynh., *Zea mays* L., *Sorghum bicolor* (L.) Moench., *Lotus corniculatus* L., and *Oryza sativa* L., further multiple sequence alignments including *LjAMT*s and AMTs from sorghum (*SbPHR*s), Arabidopsis (*AtAMT*s), and rice (*OsAMT*s) were aligned using MEGA7 (version 11.0.10). An unrooted phylogenetic tree was created using the neighbor-joining (NJ) method, with a bootstrap value of 1000. Gene structure display server program (GSDS2.0, https://gsds.gao-lab.org/, accessed on 21 February 2024) was utilized to show the exon/intron structure of the *LjAMT2;4* gene. Protein transmembrane helices prediction was performed utilizing the TMHMM Server, available online at https://services.healthtech.dtu.dk/services/TMHMM-2.0/, accessed on 21 February 2024. Three-dimensional (3D) structure modeling of LjAMT2;4 was conducted using the PyMol software (version 3.1). The *LjAMT2;4* promoter’s cis-acting elements were plotted and examined via the RSAT website (https://rsat.eead.csic.es/plants/dna-pattern_form.cgi, accessed on 21 February 2024). To further determine the similarity between *LjAMT2;4* and other reported AMTs induced by AM fungi, the protein sequences of LjAMT2;4 were compared with those of SbAMT4, GmAMT4;1, ZmAMT3;1, and SbAMT3;1 using ClustalX (version 1.81) online software, as well as Boxshade (version 3.2.3) online software.

### 2.2. Vector Construction

The p*LjAMT2;4* promoter was used to drive the expression of GUS in the pCAMBIA1301 vector. The forward primer (5′-GCAGGCATGCAAGCTTAGTGAAATTGCACCTTGCAT-3′) and reverse primer (5′-CTCAGATCTACCATGGGGCTGATGGCTTCCTTAA CC-3′) were designed to include the HindIII and NcoI restriction enzyme sites, respectively. The PCR amplification product was then ligated to produce the p*LjAMT2;4*-GUS plasmid. The *LjAMT2;4*-GFP plasmid was constructed using the subcellular localization vector pCAMBIA1305-GFP, along with the SpeI and BamHI restriction sites. These sites were integrated into the forward (5′-GGACTAGTATGTCTCTTCCCACAGCATACC-3′) and reverse (5′-CGGGATCCTAAATTTATAGTCACACCTCTT-3′) primers for PCR amplification. The restriction sites SacI and EcoRI were utilized in conjunction with the yeast expression vector pYES2, forward primer 5′-CGAGCTCATGTCTCTTCCCACAGCATACC-3′, and reverse primer 5′-GGAATTCTAAATTTATAGTCACACCTCTT-3′.

### 2.3. Subcellular Localization

The recombinant plasmid *LjAMT2;4*-GFP and the control vector were separately transformed into *Agrobacterium tumefaciens* strain GV3101, which was then infiltrated into the leaves of 5–6 week-old *Nicotiana benthamiana* plants. Following infiltration, the leaves were incubated in darkness for 48 h, and the fluorescence of the green fluorescent protein (GFP) was examined using a laser confocal microscope (Leica LSM800, Wetzlar, Germany), with excitation at 488 nm.

### 2.4. Supplementation of Ammonium-Deficient Yeast

In this study, the yeast N uptake-defective mutant 31019b (mep1Δ, mep2Δ:: LEU2, mep3Δ:: KanMX2 ura3) and expression vector pYES2 were employed. *The AtAMT1;2* and *LjAMT2;4* coding sequences were cloned into pYES2. All vectors were inserted into the yeast hexose transporter mutant 31019b using PEG/LiAc-mediated transformation [27]. Following transformation, the yeast transformants were cultured for two to three days at 30 °C on selective dropout (SC, -URA) medium supplemented with 5% maltose as the carbon source. The presence of the construct in yeast transformants was confirmed by separating and resequencing the plasmid. For complementation growth assays, the transformants were cultured in YPD liquid medium supplemented with 2% maltose overnight, washed three to five times in sterile water, and resuspended at an OD600 of 1. Subsequently, serial dilutions (10^−1^, 10^−2^, 10^−3^, and 10^−4^) were plated on YNB media containing either 2 mM arginine as a negative control or 0.02 mM, 0.2 mM, and 2 mM NH_4_^+^, respectively. *AtAMT1;2* in *A. thaliana* [28], known to rescue the phenotype of ammonium transporter-deficient yeast, was used as the positive control.

### 2.5. Experimental Materials and Planting Treatments

The arbuscular mycorrhizal fungi (AMF) species *Glomus intraradices* (Gi, provided by Sun Yat-Sen University, Guangzhou, China) and rhizobia species *Mesorhizobium loti* (lotiMAFF303099, provided by Huazhong Agricultural University), were used as fungal inoculates. Experiments were performed with *L. japonicus* LORE1-transposon insertion line (*ljamt2;4*) and wild-type (Gifu-129) plants. The sand/soil medium was mixed with vermiculite–perlite in a ratio of 4:1 and sterilized in autoclaved steam at 121 °C for 40 min. The nitrogen content of the substrate was 0.16 g/kg. Mutant (*ljamt2;4*) and wild-type (Gifu-129) plants were exposed to two ammonium concentrations (0.05 mM, 0.5 mM) to verify whether *LjAMT2;4* was affected by AMF and rhizobia and to investigate its response to varying ammonium nitrogen concentrations in both symbiotic and non-symbiotic environments. Seedlings were grown in a greenhouse at 27 °C with a 16 h light/8 h dark cycle. The plants were removed 28 days after treatment. The samples were frozen in liquid nitrogen and stored at −80 °C for subsequent RNA isolation. The culture medium for *Rhizobium* is typically TY medium, which contains yeast extract, mannitol, soil extract, and agar. The cultivation conditions are 28–30 °C, with an incubation time of 24–48 h. Prior to inoculation, the strain should undergo revival cultivation to ensure its viability and stability. Then, culture the rhizobia in liquid TY medium at 28 °C and 180 rpm overnight. When the OD600 reaches 1.0, centrifuge at 4000 rpm for 10 min to collect the rhizobia. Then, dilute the collected rhizobia with nitrogen-free medium to an OD600 of 0.05 and evenly spray the diluted rhizobia onto the roots of *L. japonicus*. The inoculation amount for each plant is 5 mL. The reproduction system of AM fungi uses a carrot root organ culture dual-culture system. Carrot roots are used as the host, and after inoculating with Gi spores on MSR medium, the culture is maintained at 25 °C. When inoculating the spores, care should be taken to keep the distance between the spores and the carrot root tips at 1–2 cm, allowing the root hairs to surround the spores. This helps shorten the time for mycelial invasion into the root hairs and accelerates the establishment of the symbiotic system. Once the spores mature, the culture medium is crushed using a juicer, and a liquid suspension is prepared via wet sieving. A 2 mL suspension containing approximately 100 spores is inoculated at the root zone of each plant of *L. japonicus.*

### 2.6. Transformation of Hairy Roots and Expression Induction by AMF

To generate p*LjAMT2;4*-GUS, a 2 kb region upstream of the *LjAMT2;4* ATG start codon was amplified via PCR from *L. japonicus* MG20 genomic DNA using Phanta Super-Fidelity DNA Polymerase (Vazyme, Nanjing, China) with primers p*LjAMT2;4*-F and p*LjAMT2;4*-R. The pCAMBIA1301 vector was used for β-glucuronidase (GUS) analysis. The pCAMBIA1301 constructs were introduced into *Agrobacterium tumefaciens* strain LBA9402 via electroporation, followed by cell cultivation on YEB solid medium. After being confirmed as positive, the colonies were moved to YMB solid medium and cultivated for two days at 28 °C. During this timeframe, *L. japonicus* seeds were sterilized (treated with 12% NaClO for 10 min, rinsed with 75% ethanol three times for 1–2 min each, followed by three to five washes with sterile water for 4–5 min each) before germination. The sterilized seeds were vernalized at 4 °C for 12 h and then spread on 1.2% water agar culture plates. Two days after germination, the seedling radicles were sectioned approximately 2 mm from the root tip using a sterile scalpel. The cut surface of the radicle was treated with *A. rhizogenes* for infection. Subsequently, the infected roots were moved to B&D medium and incubated in darkness at 28 °C for 24 h, followed by approximately three weeks of growth in a light incubator at 23 °C, with an 8 h photoperiod before transplantation. To assess the induction of the GUS gene by AMF, the expression of the gene in roots was examined by staining at 37 °C for 24 h following a 6-week symbiotic culture with AMF.

### 2.7. Detection of Mycorrhizal Colonization

The process commenced with fixing *L. japonicus* root segments in FAA for over 4 h, followed by treatment with 10% KOH and heating at 90 °C for 10 min. Subsequently, the roots were treated with a 2% HCl solution for 5 min. The root samples were stained using a 0.05% Trypan (Yeasen, Shanghai, China) blue solution and rendered transparent through treatment with lactic acid and glycerin [29].

Fifty root fragments from each 1 cm-long root sample were affixed to slides and scrutinized for specific AM structures at magnifications ranging from ×100 to ×400 using a Leica DM5000B microscope (Leica Microsystems, Wetzlar, Germany).

The statistical analysis of the mycorrhizal colonization rates was performed according to the methodology established by Trouvelot et al. (1986) [30]. The mycorrhizal colonization rate reflects AMF infection in the root segment of the host plant. Mycorrhizal colonization rate = (the number of roots infected by AMF in the sample/the total number of roots in the sample) × 100.

### 2.8. Statistical Analysis

The Student’s *t*-test was used to compare the means of the colonization rates, plant growth parameters, and relative expression levels of genes, employing the Microsoft Excel software program. All statistical data are explained in the figure legends, including the number of replicates and error bars.

## 3. Results

### 3.1. Bioinformatics Analysis of the LjAMT2;4 Gene

**Figure 1:**
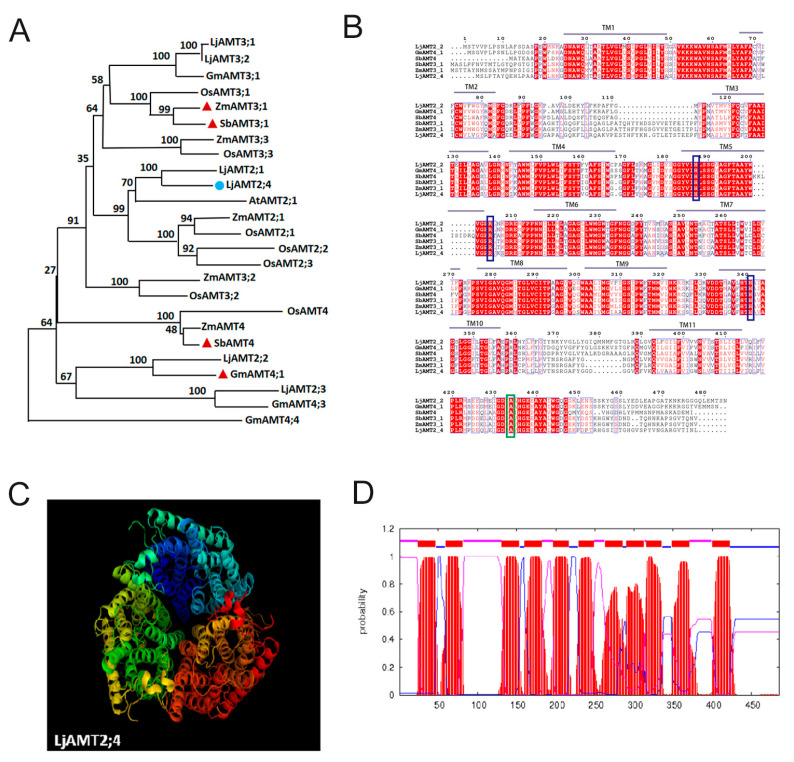
Bioinformatics analysis of *LjAMT2;4*. (**A**) Phylogenetic analysis of the AMT2 family between *L. japonicas* and other plants. The blue represents the target gene of our research, and the red represents the genes that have been reported to be upregulated by AM fungi. (**B**) Alignment of LjAMT2;4 amino acid sequence with other known AMT genes. Green lines indicate highly conserved domains, and blue lines indicate conserved ammonium transporter domains. (**C**) Predicted three-dimensional structure of LjAMT2;4 protein. (**D**) The distribution of transmembrane domains in LjAMT2;4 is depicted as follows: transmembrane regions are highlighted in red; the outer membrane is represented in blue; and the inner membrane is indicated in purple.

Ten *LjAMT* genes were identified in *L. japonicus* using the conserved ammonium transporter domain sequence as a BLASTP query in the *L. japonicus* Genomics Database. We performed multiple sequence comparisons with reported AMT genes in other plant species to construct an evolutionary tree for AMT genes. The phylogenetic tree of AMTs was divided into two clades: the AMT1 and AMT2 subfamilies. Among them, genes of the AMT2 subfamily are induced to be expressed by AM fungi *LjAMT2;2*, *ZmAMT3;1*, *SbAMT3;1*, *SbAMT4*, and *GmAMT4;1*, all of which have been reported to be upregulated by AM fungi-induced expression (Figure 1A).

Using cDNA amplification techniques, we successfully acquired the complete coding sequence of *LjAMT2;4* from the roots of wild-type *L. japonicus* (MG20). The resulting 1458 bp cDNA contained four introns that encoded a putative membrane-integrated protein of 485 amino acids.

The molecular structure of LjAMT2;4 was modeled using the PyMOL software (version 3.1). The predicted 3D model of LjAMT2; 4 features a trimeric structure typical of the ammonium transport family, with a hydrophobic pore located in the middle of each monomer (Figure 1C). The transmembrane prediction website TMHMM indicated that LjAMT2;4 has 11 transmembrane domains and is a typical membrane transporter (Figure 1D).

To further confirm the similarity between LjAMT2;4 and other reported AMTs, we compared the amino acid sequences of LjAMT2;4, SbAMT4, GmAMT4;1, ZmAMT3;1, and SbAMT3;1 using ClustalX and Boxshade. The results revealed that LjAMT2;4 has multiple and highly conserved ammonium transporter structural domains. Moreover, protein sequence similarity analysis using DNAMAN showed that LjAMT2;4 shares 73.88% amino acid sequence identity with others at the protein level (Figure 1B).

### 3.2. LjAMT2;4 Subcellular Localization

**Figure 2:**
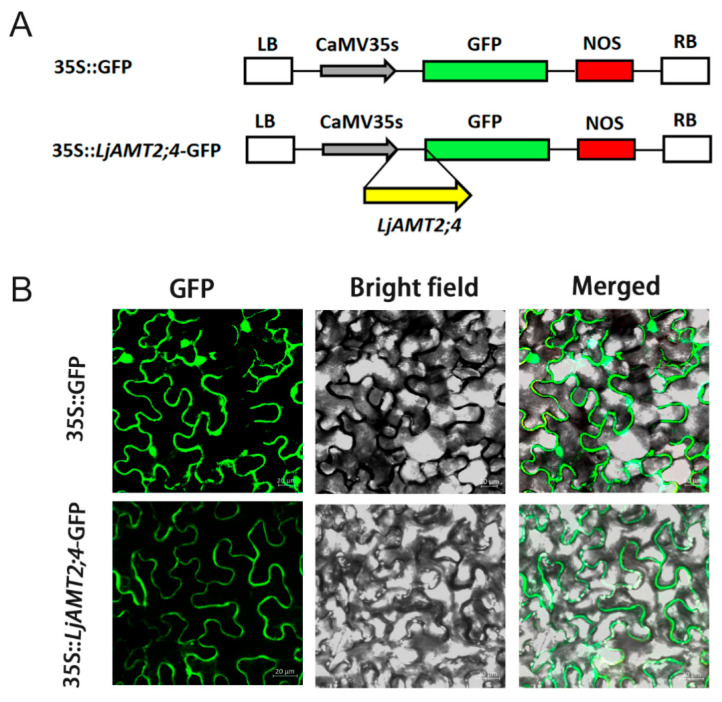
Subcellular localization of *LjAMT2;4* expression in roots. (**A**) The constructs of 35S::GFP and 35S::*LjAMT2;4*-GFP. (**B**) The target gene was 35S::*LjAMT2;4*-GFP, and the empty carrier was 35S::GFP. Three distinct visual fields were observed: the green channel (GFP), bright channel (bright field), and merged. Scale bars: 20 μM.

The subcellular localization of transporter proteins is critical for understanding mineral nutrient uptake and signal transduction in plants. To determine the subcellular localization of *LjAMT2;4*, we created a translational fusion of *LjAMT2;4* with GFP. A *LjAMT2;4*::GFP (green fluorescent protein) vector under the control of the Cauliflower mosaic virus (CaMV) 35S promoter was constructed (Figure 2A). This construction was introduced into Agrobacterium strain GV3101 (Soup-p19), along with a negative control harboring 35S::GFP. The transformed GV3101 (Soup-p19) strain was then infiltrated into *N. benthamiana* leaves. Subsequently, *N. benthamiana* leaves expressing the *LjAMT2;4*::GFP fusion protein were analyzed. Confocal imaging showed that fluorescence was observed in the plasma membrane and nucleus in the empty vector control group, whereas *LjAMT2;4*-GFP was specifically localized to the plasma membrane (Figure 2B). The green channel (GFP) is on the left, the bright field is in the center, and the merged channel is on the right-hand side.

### 3.3. Functional Analysis of LjAMT2;4 in Ammonium-Deficient Yeast

In the complementation experiment conducted to demonstrate the functionality of *LjAMT2;4*, full-length cDNA of *LjAMT2;4* was cloned into pYES2 using Gateway technology (Invitrogen). The resulting plasmids were named pYES2-*LjAMT2: 4*. The yeast (Saccharomyces cerevisiae) strain 31019b, which lacks all three endogenous NH_4_^+^ transporters (Mep1, Mep2, and Mep3), was unable to grow on a medium containing 5 mM NH_4_^+^ as the sole N source. Additionally, the low-affinity transporter *AtAMT1;2* from *A. thaliana*, known to complement the phenotype of ammonium transporter-deficient yeast, was cloned and transformed as a control [31]. Considering the acidic conditions of the peri-arbuscular space, yeast growth was analyzed using yeast nitrogen base (YNB) medium.

**Figure 3:**
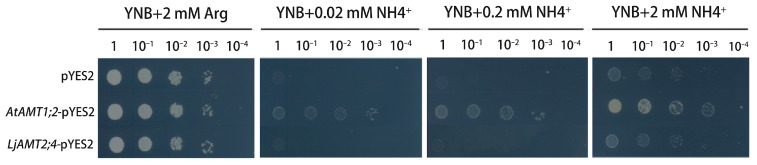
*LjAMT2; 4* functional analysis of the supplementary yeast 31019b.

As shown in Figure 3, strains transformed with pYES2 empty and *LjAMT2;4*-pYES2 vectors grew normally in YNB medium containing 2 mM arginine (0.17% YNB + 2% D-galactose + 2 mmol/L arginine + 2% agar), but did not grow normally in YNB medium containing 0.02 mM, 0.2 mM, and 2 mM ammonium ions. These results suggest that *LjAMT2;4* does not mediate ammonium transport in yeast and cannot complement the phenotype of ammonium-deficient yeast. It is possible that the effectiveness of this gene in ammonium ion transportation is limited, making it susceptible to environmental factors that may impede its functionality.

### 3.4. Tissue Expression Pattern Analysis

**Figure 4:**
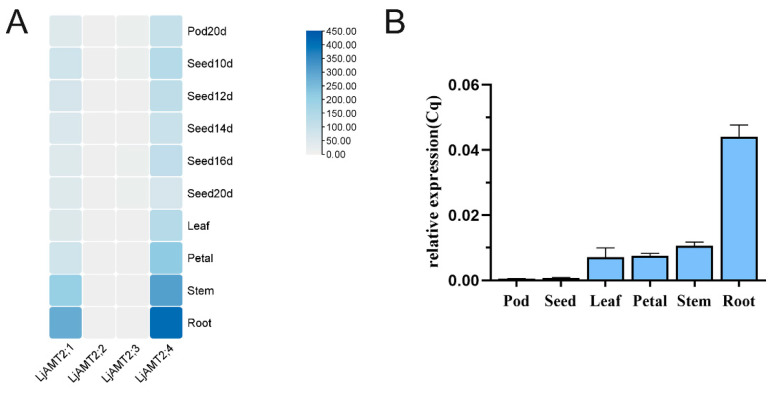
Analysis of expression patterns of *LjAMT2;4* gene. (**A**) Tissue expression pattern analysis and (**B**) qPCR results of *LjAMT2;4* expression in different tissues.

Based on the expression data from Lotus Base (https://lotus.au.dk/, accessed on 26 February 2024) and the heat map analysis conducted using TBtool (version 2.154), the expression levels of *LjAMT2;4* in the *LjAMT2* gene family were significantly higher than those of the other three genes. Specifically, *LjAMT2;4* exhibited the highest expression levels in the roots, followed by the stems, petioles, and leaves. Additionally, there was a relatively low expression of *LjAMT2;4* in seeds and pods. In contrast, *LjAMT2;2* and *LjAMT2;3* showed minimal expression in any tissue (Figure 4A). Furthermore, the qPCR results indicated that *LjAMT2;4* was mainly expressed in petals, stems, and roots, with the highest expression in roots (Figure 4B). These findings suggest that *LjAMT2;4* may play a crucial role in microbial interactions and nutrient transport processes in *L. japonicus.*

### 3.5. Identification of ljamt2;4 Mutants

**Figure 5:**
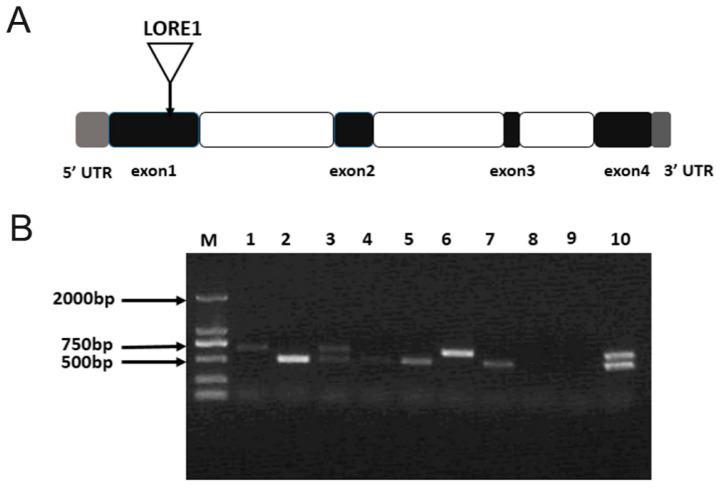
Identification of *ljamt2;4* mutants. (**A**) LORE1 insertion sites in *the ljamt2;4* mutant. Triangles represent LORE1 insertion sites: gray, 5′ UTR and 3′ UTR; white, introns; and black, exons. (**B**) Electrophoresis of genomic DNA from mutant *L. japonicus*. M represents DL2000 DNA Maker; columns 1–10 represent PCR products of mutant *L. japonicus*.

The *ljamt2;4* mutant strain originated from the Gifu background, where the LORE1 retrotransposon was inserted into exon1, resulting in a 500 bp insertion fragment. In the verification process, a three-primer method was used, with the verified mutant gene serving as the template for PCR amplification. Electrophoretic detection results showed that the target band amplified by the homozygote was a 500 bp band, the heterozygote would amplify two bands, and the wild type would appear as a large 500 bp band. According to the PCR electrophoretic diagram in Figure 5B, ten plants were verified in the experiment, and the results were verified in eight plants, including four homozygous mutant plants, two heterozygous mutant plants, and two completely wild-type plants. Homozygous plants were cultured to obtain seeds of progeny homozygous mutant traits.

### 3.6. LjAMT2;4 Negatively Regulates L. japonicus–Rhizobia Symbioses

**Figure 6:**
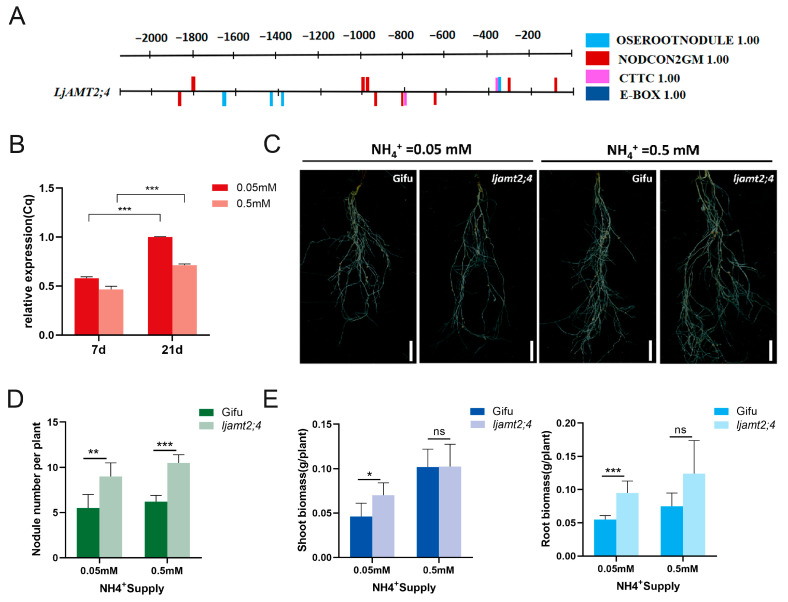
Phenotypic analysis of *ljamt2;4* symbiosis with *Rhizobium*. (**A**) Analysis of regulatory elements in the promoters of *LjAMT2;4*. (**B**) *Rhizobium*-inducible expression of the *LjAMT2;4* gene. (**C**) Phenotype of Gifu and *ljamt2;4 L. japonicas* lines post *Rhizobium* inoculation. Bars = 2 cm. (**D**) Nodule number statistics. (**E**) Statistics on shoot and root biomasses of Gifu and *ljamt2;4* post *Rhizobium* inoculation. Data are presented as the mean ± standard error (SE: n ≥ 3 biological replicates; * *p* ≤ 0.05; ** *p* ≤ 0.01; *** *p* ≤ 0.001 and ns: no significant difference).

To further explore whether the *LjAMT2;4* gene sequence may be induced by AM fungi or rhizobia, we analyzed the promoter sequence of the upstream 2000 bp. The promoter online analysis website RSAT indicates that it contains multiple rhizobia-responsive cis-acting elements, NODCON2GM (CTCTT) and OSEOOTNODULE(AAAGAT), as well as two arbuscular mycorrhizal-responsive cis-acting elements CTTC (Figure 6A).

Next, we selected two important nodes of the rhizobia symbiosis pathway for the rhizobia-induced expression analysis of *LjAMT2;4*. After inoculation with rhizobia, it was positively distributed in the roots with an increase in inoculation days. The expression of *LjAMT2;4* in the roots inoculated with rhizobia at 21 days was significantly higher than that at 7 days (Figure 6B). This may be due to the fact that nodule primordia are formed at 7 dpi, and organogenesis is progressing, and at 21 dpi, when most of the nodules are mature, symbiont differentiation is complete and symbiotic nitrogen fixation is established. In addition, the expression level at a concentration of 0.05 mM was greater than that at 0.5 mM. This indicates that *LjAMT2;4* is induced by rhizobia, and that its expression is significantly induced under nitrogen starvation conditions.

These results provide a basis for further research on the involvement of *LjAMT2;4* in rhizobia symbiosis. To further explore the regulation of this gene involved in the symbiotic pathway, we inoculated *ljamt2;4* and the wild-type control with *M. loti*. After 6 weeks, the results showed that the number of nodules in the mutant was higher than that in the wild-type line (Figure 6C). The number of nodules increased by 59.6% and 60.1% at 0.05 mM and 0.5 mM ammonium concentrations, respectively (Figure 6D). This suggests that this gene may regulate effectors associated with symbiotic nodules. In the extreme *N*. *benthamiana* nitrogen starvation environment (0.05 mM), the above-ground and underground biomasses of the mutant lines were higher than those of the wild type, and there was no significant difference between the two under mild N starvation (0.5 mM) (Figure 6E).

In summary, compared with the wild type, *ljamt2;4* significantly increased the nodule number, suggesting that this gene may be involved in rhizobia symbiotic signaling. In contrast, *ljamt2;4* promoted its own growth under extreme nitrogen starvation. This suggests that symbiotic nitrogen fixation can effectively alleviate the survival needs of plants in a nitrogen starvation environment and is the most important source of sustainable agricultural nitrogen fertilizer.

### 3.7. Mycorrhizal-Inducible Expression of the LjAMT2;4 Gene

**Figure 7:**
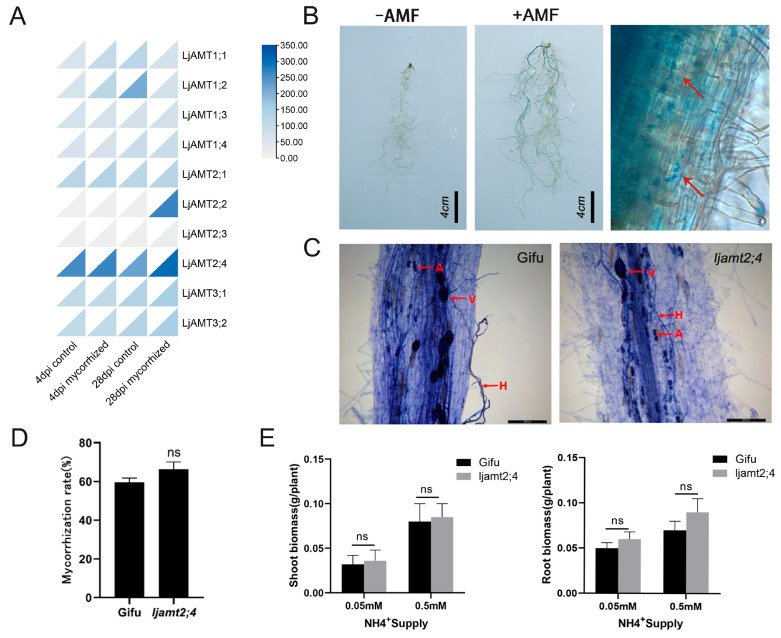
Mycorrhizal-inducible expression of *LjAMT2;4*. (**A**) Heatmap of *LjAMT* family gene expression induced by AMF. (**B**) Detection of GUS expression induced by AM fungi in p*LjAMT2; 4*. Arrows indicate GUS expression positions. Scale bar: 4 cm. (**C**) Trypan blue staining of Gifu and *ljamt2;4* after inoculation with *R. irregularis*. H, hyphae; V, vesicle; A, arbuscule. Scale bar: 200 μm. (**D**) Mycorrhization rate statistics. (**E**) Statistics on shoot and root biomasses of Gifu and *ljamt2;4* post *R. irregularis* inoculation. Data are presented as the mean ± standard error (SE: n ≥ 3 biological replicates; ns: no significant difference).

In a 2009 paper by Guether et al., transcriptome data were detected at 4 d and 28 d after symbiosis between the AMF of *L. japonicus*, as well as in non-symbiotic conditions. By drawing the expression heat map, it was found that *LjAMT2;4* had the highest expression level among all the genes (Figure 7A). The expression of *LjAMT2;4* was slightly higher than that of the control at 28 days after AM fungi symbiosis.

To further investigate the potential induction of *LjAMT2;4* by AM fungi, we isolated a 2 kb upstream sequence of *LjAMT2;4* and fused it to the GUS reporter gene (to generate the p*LjAMT2;4*::GUS plasmid). The promoter fusion GUS expression vector was transformed into *Agrobacterium tumefaciens* strain LBA9402, which was transformed into the hairy roots of *L. japonicus* using *Agrobacterium tumefaciens* infestation and cultured in symbiosis with arbuscular mycorrhizal (AM) fungi. After 6 weeks, transformed mycorrhizae were stained blue with X-Gluc in the colonized root area of p*LjAMT2;4*::GUS-transformed hairy roots, while no blue color was observed in non-colonized transformed *L. japonicus* hairy roots (Figure 7B). The roots of *L. japonicus* after AM fungal symbiosis were observed under a microscope. Microscopic examination of *L. japonicus* roots following AM fungal symbiosis revealed that GUS expression predominantly occurred within the vascular column and endothelial cells adjacent to the vascular column, with comparatively minimal expression in the outer cortex cells (Figure 7B). These results indicate that *LjAMT2;4* expression is induced by AM fungi.

To further investigate the response of *LjAMT2;4* to arbuscular mycorrhizal fungi (AMF), we conducted inoculation experiments with *G*. *intraradices* on both *ljamt2;4* mutant and wild-type control plants. After five weeks of symbiosis, trypan blue staining of the roots of the wild type and *ljamt2;4* showed that both had a large number of hyphae, arbuscules, and vesicles (Figure 7C). This showed that both the experimental and control groups met the symbiosis requirements. However, the insertion line ljamt2;4 showed a small increase in mycorrhizal colonization levels compared to wild-type plants, but the difference was not significant (Figure 7D).

Furthermore, to investigate the effect of N concentration on the growth of *L. japonicus*, the shoot and root biomasses of Gifu and *ljamt2;4* under different ammonium concentrations and inoculated with Gi were measured. There was no significant difference between *ljamt2;4* and the wild type under any nitrogen conditions (Figure 7E).

In conclusion, these findings suggest that *ljamt2;4* has no significant effect on the phenotype of *L. japonicus* in a symbiotic environment. This suggests that although *LjAMT2;4* is induced by AM fungi, its role in symbiosis with AM fungi is weak. Furthermore, it indicates that *LjAMT2;4* does not have the ability to transport ammonium nitrogen.

## 4. Discussion

In this study, we identified the characteristics of the ammonium transporter *LjAMT2;4* in *L. japonicus*. This transporter was found to be localized in the plasma membrane and mainly expressed in roots. Additionally, it contains multiple rhizobia-responsive cis-acting elements, NODCON2GM(CTCTT) and OSEOOTNODULE(AAAGAT), as well as two arbuscular mycorrhizal-responsive cis-acting elements CTTC.

In the past, it was often reported that the AMT2 subfamily is induced to be expressed by AM fungi, and *LjAMT2;2*, *ZmAMT3;1*, *SbAMT3;1*, *SbAMT4*, and *GmAMT4;1* have all been reported to be upregulated by AM fungi-induced expression [13,32,33,34]. Similarly, in our experiment, transformed mycorrhizae were stained a distinct blue with X-Gluc stain in the colonized root area of p*LjAMT2;4*::GUS-transformed hairy roots, while no blue color was observed in non-colonized transformed *L. japonicus* hairy roots. This is consistent with the findings of Kobae et al. (2010) [12]. Cells containing p*GmAMT4;1*-GUS displayed consistent GUS activity in endodermal cells, indicating that the promoter of this gene was induced by AMF. However, the insertion line *ljamt2;4* did not differ significantly from the wild type in terms of growth, and there was only a small increase in the level of mycorrhizal colonization compared to in wild-type plants.

Interestingly, the previous symbiosis heatmap showed that *LjAMT2;2* was significantly upregulated at 28 days by AM fungi-induced expression. A previous study reported that the overexpression of *LjAMT2;2* after symbiosis with AM fungi promoted the growth and development of *L. japonicus* and, to a certain extent, facilitated the uptake of ammonium nitrogen in *L. japonicus* [35]. Therefore, we speculate that this may be due to the closer affinity of *LjAMT2;4* to *LjAMT2;2* in *L. japonicus*, resulting in the functional redundancy of *LjAMT2;4*.

Until now, it has been reported that the promotion of AM symbiosis in the rhizosphere microbial community promotes the nodulation of different legumes [36]. As legumes can establish symbiotic relationships with both arbuscular mycorrhizal (AM) fungi and rhizobia, and given the presence of multiple rhizobia-responsive elements identified in the previous promoter analysis, we further investigated the functional impact of the *LjAMT2;4* gene on rhizobia symbiosis. Our experiments revealed that the *ljamt2;4* mutant exhibited a significant increase in number of nodules compared to the wild type under various N-starved conditions. In particular, in an extreme nitrogen starvation environment, the *ljamt2;4* biomass was considerably higher than that of the wild type, especially in the underground part. This suggests that *LjAMT2;4* negatively regulates nodule number and plays an important role in mycorrhizal symbiotic relationships. *L. japonicus* effectively fixes nitrogen in the air by generating nodules in symbiosis with rhizobia, and releases nitrogen from rhizomes for plant growth under extreme nitrogen starvation conditions. Symbiotic nitrogen fixation can effectively alleviate the survival needs of plants in a nitrogen starvation environment and is the most important source of sustainable agricultural nitrogen fertilizer. These findings have rarely been reported in the literature. Little is currently known about the genes that regulate symbiotic nitrogen fixation. Therefore, our study provides a possible basis for the use of genes to regulate symbiotic nitrogen fixation and offers new ideas for the sustainable development of agricultural production.

Legumes are host plants that can form symbiotic relationships with both AM fungi and rhizobia. A key trait of legumes is their competence for symbiotic nitrogen fixation, which is the result of an intimate relationship with a group of soil-living bacteria, collectively called rhizobia [37]. Rhizobia symbiotically fix nitrogen in host plants through four major processes: recognition, infestation, nodulation, and nitrogen fixation [18]. Under symbiotic treatment with rhizobia, the number of *ljamt2;4* nodules significantly increased compared with in the wild type, indicating that this gene may be involved in rhizobia symbiotic signaling. However, the reasons for the negative regulation of the nodule number by *LjAMT2;4* and the mechanisms of symbiotic nitrogen fixation with rhizobia remain to be elucidated. Ertao Wang’s team discovered that SCR, an important regulator of root stem cells, is conserved and specific in legume root nodule development, and that the overexpression of SHR-SCR can induce root cortex cell division [38]. Research has found that the cell surface receptor *SYMRK* (symbiosis receptor kinase) is responsible for mediating symbiotic signals, from rhizobia perception to nodule formation [39,40,41,42]. In *L. japonicus*, the gene *SYMRKL1* encodes a protein featuring a domain that closely resembles *SYMRK*, which is essential for the formation of infestation lines [26,43]. Does *LjAMT2;4* play an important role in this process, from the formation of infection threads to the generation of nodules? In addition, *LjIMA1* and *LjIMA2* were found to concentrate iron in the nodules at the stem and root of *L. japonicus*. IMA peptides play a conservative role in nitrogen homeostasis regulation by modulating the nitrogen–iron balance in both *A. thaliana* and *L. japonicus* [44]. Whether *LjAMT2;4* is linked to these genes remains to be proven by further studies.

Furthermore, the fact that *LjAMT2;4*, a typical ammonium transporter protein, cannot directly and efficiently transport ammonium ions is an issue that deserves further investigation. There is no cis-acting E-BOX associated with ammonium nitrogen transport in the promoter region of *LjAMT2;4*, suggesting that *LjAMT2;4* does not effectively transport ammonium ions. Simultaneously, there is a possibility that the efficacy of this gene in ammonium ion transportation is limited, rendering it vulnerable to environmental factors that could hinder its functionality. In addition, *MtAMT2;3* does not transport ammonium, but acts as an ammonium sensor to regulate the development of arbuscules, thus affecting the acquisition of symbiotic nutrients (N and P) [16,43,45]. It is plausible that *LjAMT2;4* has evolved alternative pathways for symbiotic nitrogen fixation during mycorrhizal symbiosis, because of its inherent inability to directly and effectively transport ammonium ions.

## 5. Conclusions

This study focuses on the leguminous model plant *L. japonicus* and systematically analyzes the molecular characteristics of the ammonium transporter *LjAMT2;4* and its regulatory role in the plant–microbe symbiotic system. The results show that the expression of *LjAMT2;4* is induced by rhizobia (*M. loti*) and arbuscular mycorrhizal fungi (*G. intraradices*). Under 0.5 mM and 0.05 mM nitrogen-stress conditions, this gene significantly improved nodule formation efficiency (by approximately 59.6% and 60.1%, respectively) by promoting symbiosis with rhizobia, thereby enhancing biological nitrogen fixation and promoting plant growth. However, in the AM fungal symbiotic system, no significant differences were observed in the mycorrhizal infection rate or plant biomass between wild-type and *LjAMT2;4* mutant plants (*p* > 0.05). Previous studies have reported that *LjAMT2;2* regulates the symbiosis between AM fungi and *L. japonicus*, suggesting that *LjAMT2;4* may exhibit functional redundancy in the AM symbiotic pathway. Given the important role of rhizobia and AM fungal symbiotic systems in agricultural ecosystems, this study provides new theoretical insights for understanding plant–microbe interaction mechanisms and offers important references for developing sustainable agricultural management strategies based on microbial symbiosis.

## Data Availability

The original contributions presented in this study are included in the article. Further inquiries can be directed to the corresponding authors.
